# Supplementary material for: A Comprehensive Assessment of Ultraviolet-Radiation-Induced Mutations in Flammulina filiformis Using Whole-Genome Resequencing
Source: J Fungi (Basel). 2024 Mar 20;10(3):228. doi: 10.3390/jof10030228 (PMC10971301; doi:10.3390/jof10030228)
Supplement: Supplementary file 1 [file jof-10-00228-s001.zip › Supplementary Material S8/KEGG annotation/out/64381550635650.os/KO/out_map/map04922.html]

KEGG PATHWAY: Glucagon signaling pathway - Reference pathway


|  |  |
| --- | --- |
| **Glucagon signaling pathway - Reference pathway** |  |

[
Pathway menu
| Organism menu
| Pathway entry
| Show description
| User data mapping
]

Glucagon is conventionally regarded as a counterregulatory hormone for insulin and plays a critical anti-hypoglycemic role by maintaining glucose homeostasis in both animals and humans. To increase blood glucose, glucagon promotes hepatic glucose output by increasing glycogenolysis and gluconeogenesis and by decreasing glycogenesis and glycolysis in a concerted fashion via multiple mechanisms. Glucagon also stimulates hepatic mitochondrial beta-oxidation to supply energy for glucose production. Glucagon performs its main effect via activation of adenylate cyclase. The adenylate-cyclase-derived cAMP activates protein kinase A (PKA), which then phosphorylates downstream targets, such as cAMP response element binding protein (CREB) and the bifunctional enzyme 6-phosphofructo-2-kinase/ fructose-2,6-bisphosphatase (one of the isoforms being PFK/FBPase 1, encoded by PFKFB1).

|  |  |
| --- | --- |
| Reference pathway | 100% |
